# Resistome Profiles, Plasmid Typing, and Whole-Genome Phylogenetic Tree Analyses of *Bla*_NDM-9_ and *Mcr-1* Co-Harboring *Escherichia coli* ST617 from a Patient without a History of Farm Exposure in Korea

**DOI:** 10.3390/pathogens8040212

**Published:** 2019-10-31

**Authors:** Le Phuong Nguyen, Naina Adren Pinto, Thao Nguyen Vu, Hung Mai, An HT Pham, Hyunsook Lee, Young Lag Cho, Jung-Hyun Byun, Roshan D’Souza, Dongeun Yong

**Affiliations:** 1 Department of Laboratory Medicine and Research Institute of Bacterial Resistance, Yonsei University College of Medicine, Seoul 03722, Korea; luongphekidz07@gmail.com (L.P.N.); naina.pinto@gmail.com (N.A.P.); vuthaonguyen1992@gmail.com (T.N.V.); snoopycat78@gmail.com (H.L.); roshanbernard@gmail.com (R.D.); 2 Brain Korea 21 PLUS Project for Medical Science, Yonsei University, Seoul 03722, Korea; 3 Faculty of Pharmacy, University of Debrecen, Debrecen 4032, Hungary; mhmaihung@gmail.com (H.M.); phamhuynhthuyan@gmail.com (A.H.P.); 4 Legochem Biosciences, Daejeon 34302, Korea; young@legochembio.com; 5 Department of Laboratory Medicine, Gyeongsang National University Hospital, and Gyeongsang National University College of Medicine, Jinju 52727, Korea; microbyun@gmail.com; 6 J. Craig Venter Institute, Rockville, MD 20850, USA

**Keywords:** *bla*_NDM-9_, co-harboring *Escherichia coli*, *mcr-1*

## Abstract

Recently, a *bla*_NDM-9_ and *mcr-1* co-harboring *E. coli* ST 617 isolate was identified from an asymptomatic carrier in Korea. An 81-year-old female was admitted to a university hospital for aortic cardiac valve repair surgery. Following surgery, she was admitted to the intensive care unit (ICU) for three days, and carbapenem-resistant *E. coli* YMC/2017/02/MS631 was isolated from a surveillance culture (rectal swab). Antimicrobial susceptibility testing (AST) for colistin was not performed at that time. Upon retrospective study, further AST revealed resistance to all tested antibiotics, including meropenem, imipenem, ceftazidime-avibactam, amikacin, gentamicin, ciprofloxacin, trimethoprim-sulfamethoxazole, and colistin, with the exception of tigecycline. Whole genome sequencing analyses showed that this strain belonged to the ST617 serotype O89/162: H10 and harbored three *β*-lactamase genes (*bla*_TEM-1B_, *bla*_CTX-M-55_, *bla*_NDM-9_), mcr-1, and 14 other resistance genes. Seven plasmid replicon types (IncB, IncFII, IncI2, IncN, IncY, IncR, IncX1) were identified. Horizontal transfer of *bla*_NDM-9_ and *mcr-1* from donor cells to the recipient *E. coli* J53 has been observed. *bla*_NDM-9_ and *mcr-1* were carried by IncB and IncI2 plasmids, respectively. To speculate on the incidence of this strain, routine rectal swab screening to identify asymptomatic carriers might be warranted, in addition to the screening of ICU patients.

## 1. Introduction

Since the first New Delhi metallo-*β*-lactamase 1 (*bla*_NDM-1_) was identified from a carbapenem-resistant *Klebsiella pneumoniae* strain [1], several variations of this metallo-β-lactamase have been discovered all over the world. One variant, *bla*_NDM-9_, was identified from a *K. pneumoniae* clinical isolate in China [2]. Accompanying the rise of carbapenem-resistant bacteria, colistin has become used more commonly, despite its serious side effects. Under the selective pressure of colistin, the first *bla*_NDM-9_ and *mcr-1* co-harboring *E. coli* ST167 strain was identified from a retail chicken in 2016 [3]. Subsequent thereto, *E. coli* strains co-harboring *bla*_NDM-9_ and *mcr-1* were reported from a chicken farm in China [4]. Since then, there have been concerns over the spread of this strain to humans. A *bla*_NDM-9_ and *mcr-1* co-harboring *E. coli* 5CRE51 strain from a urine sample was first reported in Taiwan in 2017 [5]. This strain carried two different plasmids that harbored *bla*_NDM-9_ and *mcr-1* and belonged to the ST617 strain [6]. Recently, a *bla*_NDM-9_ and *mcr-1* co-harboring *E. coli* isolate was identified from an asymptomatic carrier in Korea in a retrospective study. Subsequent thereto, phenotypic and phylogenomic analyses were conducted to trace the strain’s origin.

## 2. Case Report

An 81-year-old female was admitted to a university hospital in February 2017 for aortic cardiac valve repair surgery. The patient had chest pain and dyspnea on exertion that had been aggravated six months before a diagnosis of severe aortic regurgitation. The patient had no history of abroad travel, visiting a farm or any signs of infections. She received preoperative antibiotic prophylaxis with amoxicillin-clavulanate. Following surgery, she was admitted to the intensive care unit (ICU) for three days, and carbapenem-resistant *E. coli* YMC/2017/02/MS631 was isolated from a rectal swab surveillance culture, which was routinely performed to stop the spreading of *Klebsiella pneumoniae* carbapenemase-producing Enterobacteriaceae in the ICU. Antimicrobial susceptibility testing (AST) for colistin was not performed at that time. Upon retrospective study, colistin resistance was detected: In vitro AST was performed using both broth microdilution and agar dilution methods. The results were interpreted according to the Clinical and Laboratory Standards Institute guidelines [7], with the exception of tigecycline and colistin, for which the European Committee on Antimicrobial Susceptibility Testing v9.0 was applied (http://www.eucast.org/clinical_breakpoints/). The isolate was resistant to all tested antibiotics (amoxicillin-clavulanic acid, piperacillin, piperacillin-tazobactam, cefotaxime, ceftazidime, cefepime, cefoxitin, aztreonam, ertapenem, meropenem, imipenem, ceftazidime-avibactam, amikacin, gentamicin, ciprofloxacin, trimethoprim-sulfamethoxazole, and colistin), except for tigecycline (Table 1). 

Whole genomic DNA was extracted and sequenced using an Illumina Hiseq2500^TM^ (Illumina, Valencia, CA, USA). Sequence reads were assembled using SPAdes version 3.12 [8]. The genome was annotated employing Rapid Annotation using Subsystem Technology [9] and deposited in the NCBI GenBank with the accession number SBHK00000000. Resistance genes, serotype, multi-locus sequence type (MLST), plasmid replicon type, and virulence factors were analyzed using ResFinder 3.1 [10], SeroTypeFinder 2.0 [11], MLST 2.0 [12], PlasmidFinder 2.0 [13], and VirulenceFinder 2.0 [14], respectively. Despite sharing the same sequence type (ST617) and serotype (O89/162: H10) with *E. coli* 5CRE51 [6], this strain carried additional resistance genes, namely, *bla*_TEM-1B_, *bla*_CTX-M-55_, *aph(3’)IIa, aph(3’)Ib, rmtB, aph(6)-Id, oqxA, oqxB, mph(A), mdf(A),* and *sul2,* accounting for cephalosporin, aminoglycoside, fluoroquinolone, macrolide, and sulfamethoxazole resistance (Table 1). Additionally, the *mcr-1* sequence from *E. coli* YMC/2017/02/MS631 had one silent mutation at position 1074 (C→A), compared with a previously reported *mcr-1*. Seven plasmid replicon types (IncB, IncFII, IncI2, IncN, IncY, IncR, IncX1), and two virulence factors (*gad*, *iss*) were identified. Transferability of the *bla*_NDM-9_ and *mcr-1* genes was achieved by conjugation experiments with *E. coli* J53 as the recipient strain [15,16]. Transconjugants were selected on Mueller–Hinton agar plates containing 100 μg/mL of sodium azide with 4 μg/mL of imipenem or 2 μg/mL of colistin. Resistance genes and plasmid replicon types in the transconjugants were identified by PCR and Sanger sequencing using designed primers (Table S1). The transconjugant *E. coli* EJ533 was susceptible to all tested antibiotics but colistin and harbored *mcr-1* on the IncI2 type plasmid. Meanwhile, the transconjugant *E. coli* EJ5331 was resistant against all β-lactams but aztreonam and carried *bla*_NDM-9_ and other resistance genes (*aadA2*, *fosA3, mph(A), sul2, and dfrA12)* on the IncB type plasmid (Table 1). 

**Figure 1:**
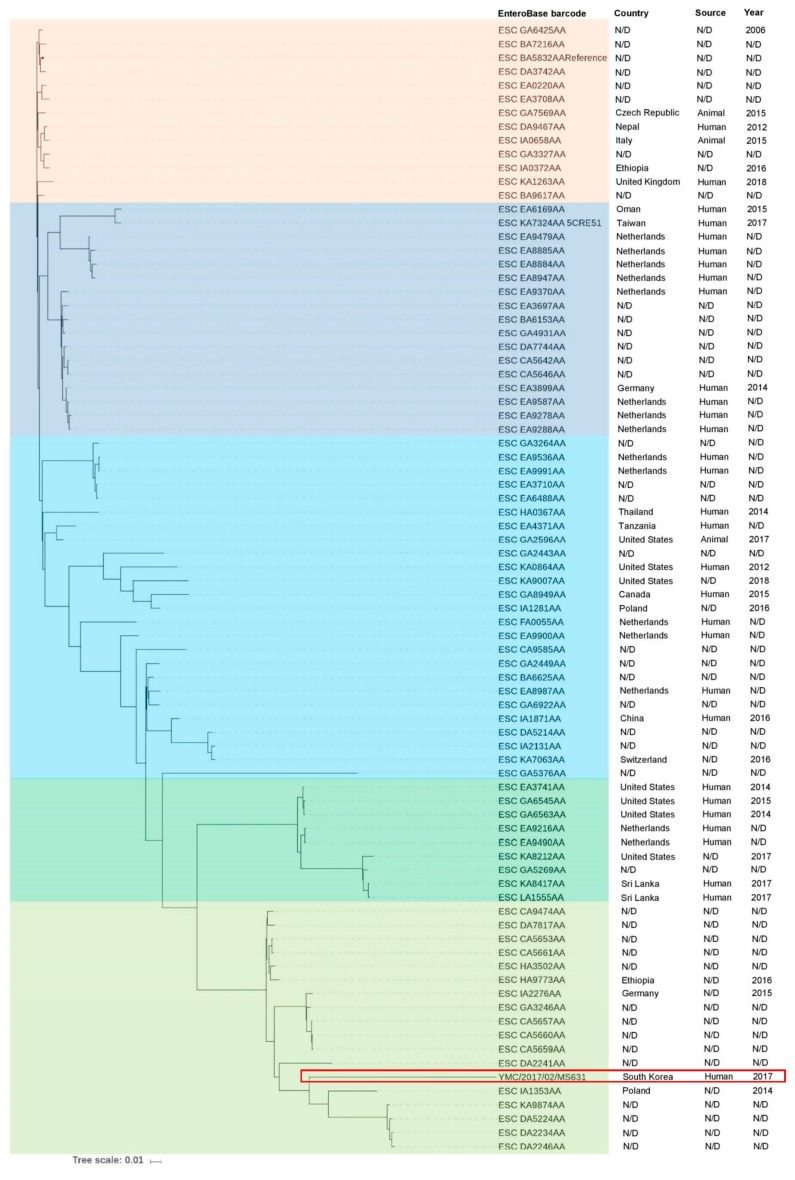
Phylogenomic comparison of the 82 *E. coli* ST617 strains collected from EnteroBase (http://enterobase.warnick.ac.uk/). The phylogenetic tree was constructed using CSI phylogeny 1.4 (https://cge.cbs.dtu.dk/services) with the standard parameters. iTOL (https://itol.embl.de/) was used to visualize the phylogenetic tree. The strain ESC_BA5832AA was selected as the reference strain. Origin, source and collection year of the isolates also were illustrated. The red box indicates the *E. coli* YMC/2017/02/MS631, which is closely related to the strain ESC_IA1353AA isolated from Poland in 2014.

The relatedness of *E. coli* 5CRE51 and YMC/2017/02/MS631 in the context of worldwide *E. coli* ST617 distribution was investigated using EnteroBase database (http://enterobase.warnick.ac.uk/) (Figure 1). Altogether, 200 *E. coli* ST617 strains among 82,737 *E. coli* genomes (0.24%) have been reported in EnteroBase with the key words “*E. coli*” and “ST617” at the time of writing this paper (File S1). *E. coli* ST617 strains have been detected in at least 31 countries and from different sources, including human, animal, and environmental samples (Figure S1, S2A). Among them, 170 *E. coli* genomes were accessible for analysis (File S1). Thirteen different serotypes were identified from the collected genomes. Considering 170 genomes, 82 (48.2%) belonged to serotype O89/162: H10 (Figure S2B) and were included in the phylogenomic tree constructed using CSI phylogeny 1.4 [17]. The phylogenetic tree was visualized using iTOL (https://itol.embl.de/) and indicated that *E. coli* YMC/2017/02/MS631 was distant from *E. coli* 5CRE51 and other strains. The closest strains were detected in Poland (ESC_IA1353AA) and Germany (ESC_IA2276AA) (Figure 1). Meanwhile, *E. coli* 5CRE51 belonged to a clade with strains collected from Oman (ESC_EA6169AA) and The Netherlands (ESC_EA9479AA). This suggested that the two strains emerged independently. 

## 3. Discussion

The first *bla*_NDM-9_ and *mcr-1* co-harboring *E. coli* isolate belonged to a ST167 strain was followed by ST10 complexes, ST101, ST156, and ST297 [3,4]. The primary source was chicken meat. This strain rarely has been reported in clinical settings. Our report is important because it was detected coincidentally in a retrospective cohort study, and the strain displayed high antibiotic resistance with complicated plasmid types. Additionally, this strain displayed a broader resistance spectrum than the *E. coli* 5CRE51 isolate from Taiwan. Furthermore, the strain had two- and eight-folds higher minimal inhibitory concentrations (MICs) for meropenem and imipenem and an eight-fold higher MIC for amikacin in comparison with *E. coli* 5CRE51. However, the colistin MIC was the same: 4 μg/mL [5]. Resistome profiles, plasmid typing, and whole-genome phylogenetic tree analyses suggested that the two strains were not closely related. Interestingly, *E. coli* ST617 has mainly been detected in European countries, the United States, and China, and only two isolates of *E. coli* ST617 have been reported for Taiwan and Korea, according to EnteroBase (Figure S2A). The dominance of E. coli ST617 serotype O89/162:H10 among 13 serotypes may suggest that *E. coli* ST617 serotype O89/162: H10 can be a potential reservoir for *bla*_NDM-9_ and *mcr-1* co-harboring *E. coli* in clinical settings.

According to the EUCIC medical guidelines on decolonization [18], there is a lack of evidence regarding the efficiency of decolonization for multidrug-resistant gram-negative organisms in hospitalized patients. Hence, the patient can be discharged without any treatment or intervention for decolonization. This may, in turn, lead to a silent continuous spread of this strain in the population at large. Additionally, travel by asymptomatic *bla*_NDM-9_ and *mcr-1* carriers could facilitate strain transmission. A recent study emphasized the contribution of fecal pollution to an abundance of resistance genes in effluent-receiving environments [19]. Routine rectal swab screening to identify asymptomatic colistin and/or carbapenem-resistance carriers might be warranted to control the spread of multi-drug resistant organisms in hospital settings. 

## 4. Conclusions

To the best of our knowledge, this is the first report of resistome profiles, plasmid typing, and whole-genome phylogenetic tree analyses of a *bla*_NDM-9_ and *mcr-1* co-harboring *E. coli* strain isolated from an asymptomatic carrier. Moreover, the resistance genes were transferable to azide-resistant *E. coli* J53. Further surveillance studies should be conducted to detect the prevalence of this strain among health-care systems in Korea.

## Figures and Tables

**Table 1:** Characterization of *mcr-1*, *bla*_NDM-9_, and *bla*_CTX-M-55_-positive *E. coli* YMC/2017/02/MS631 and its transconjugants.

Parameters	*E. coli* YMC/2017/ 02/MS631	Transconjugants		
		Selected by Colistin	Selected by Imipenem	*E. coli* J53
		*E. coli* EJ533	*E. coli* EJ5331	
Source	Asymptomatic carrier	-	-	-
Isolation site	Rectal swab	-	-	-
Resistance genes	*bla*_NDM-9_, *bla*_CTX-M-55_, *bla*_TEM-1B_, *aph(3’)IIa, aph(3’)Ib, rmtB, aph(6)-Id, aadA2, oqxA, oqxB, fosA3, mph(A), mdf(A),floR, sul2, tet(A), dfrA12, mcr-1*	*mcr-1*	*bla*_NDM-9_, *aadA2*, *fosA3, mph(A),* *dfrA12*	-
MLST	617	-	-	-
Serotype	O89/162:H10	-	-	-
Plasmid replicon type(s)	IncB, IncFII, IncI2, IncN, IncY, IncR, IncX1	IncI2	IncB	-
Virulence factors	*gad*, *iss*			
*ompC, ompF*	Intact			
MIC (μg/mL, interpretation)				
Amoxicillin-clavulanic acid	128, R ^†^	4, S ^‡^	8, R ^‡^	4, S ^‡^
Piperacillin	≥256, R ^†^	N/D	N/D	N/D
Piperacillin-tazobactam	≥256, R ^†^	≤4, S ^‡^	≥128, R^‡^	≤4, S ^‡^
Cefotaxime	≥256, R ^†^	≤1, S ^‡^	≥64, R ^‡^	≤1, S ^‡^
Ceftazidime	≥256, R ^†^	≤1, S ^‡^	≥64, R ^‡^	≤1, S ^‡^
Cefepime	≥256, R ^†^	≤1, S ^‡^	≥64, R ^‡^	≤1, S ^‡^
Cefoxitin	≥256, R ^†^	8, S ^‡^	32, R ^§^	≤1, S ^‡^
Aztreonam	≥128, R ^†^	≤1, S ^‡^	≤1, S ^‡^	≤1, S ^‡^
Ertapenem	64, R ^†^	≤0.5, S ^‡^	4, R ^‡^	≤0.5, S ^‡^
Meropenem	16, R ^†^	N/D	N/D	N/D
Imipenem	32, R ^†^	≤0.25, S ^‡^	8, R ^‡^	≤0.25, S ^‡^
Ceftazidime-avibactam	≥256, R ^†^	N/D	N/D	N/D
Colistin	4, R ^‡^	4, R ^‡^	≤0.125, S ^‡^	<0.125, S ^‡^
Amikacin	≥16, R ^‡^	≤2, S ^‡^	≤2, S ^‡^	≤2, S ^‡^
Gentamicin	≥16, R ^‡^	≤1, S ^‡^	≤1, S ^‡^	≤1, S ^‡^
Ciprofloxacin	≥4, R ^‡^	≤0.25, S ^‡^	≤0.25, S ^‡^	≤0.25, S ^‡^
Tigecycline	0.5, S ^‡^	≤0.5, S ^‡^	≤0.5, S ^‡^	≤0.5, S ^‡^
Trimethoprim-sulfamethoxazole	320, R ^‡^	≤20, S ^‡^	≤20, S ^‡^	≤20, S ^‡^

Abbreviations: MIC, minimal inhibitory concentration; MLST, Multi Locus Sequence Type; N/D, not determined. In vitro antimicrobial susceptibility testing was performed using an agar dilution method † and a broth microdilution method ^‡^ following the Clinical and Laboratory Standards Institute (CLSI) guidelines M100 28th ed. MIC interpretations followed CLSI M100 28th ed, with the exception of tigecycline and colistin, for which the European Committee on Antimicrobial Susceptibility Testing guidelines v9.0 were applied.

## Data Availability

The genome YMC/2017/02/MS631 was deposited in the NCBI GenBank with the accession number SBHK00000000.

## Supplementary Materials

The following are available online at https://www.mdpi.com/2076-0817/8/4/212/s1.

## Abbreviations

AST: antimicrobial susceptibility testing; EUCIC: European Committee on Infection Control; ICU: Intensive care unit; MIC: Minimal inhibitory concentration; NCBI: National center for biotechnology information.
