# Supplementary material for: Resistome Profiles, Plasmid Typing, and Whole-Genome Phylogenetic Tree Analyses of BlaNDM-9 and Mcr-1 Co-Harboring Escherichia coli ST617 from a Patient without a History of Farm Exposure in Korea
Source: Pathogens. 2019 Oct 31;8(4):212. doi: 10.3390/pathogens8040212 (PMC6963575; doi:10.3390/pathogens8040212)
Supplement: Supplementary file 1 [file pathogens-08-00212-s001.zip › Supplementary data Pathogen Figure S1.pdf]

Supplementary Data:

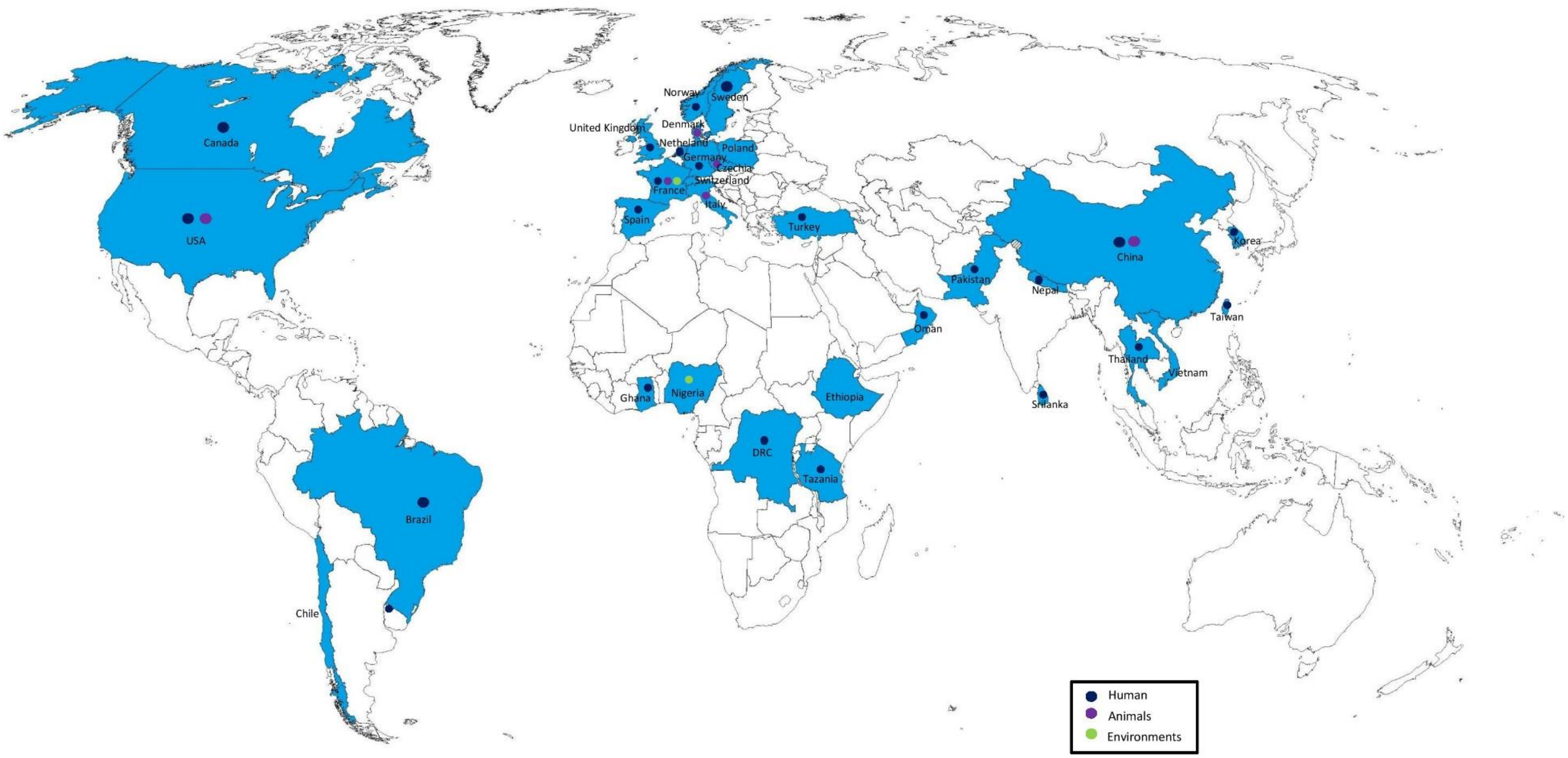

Figure S1. Global distribution of *E. coli* ST617. The countries where *E. coli* ST617 has been detected are coloured in blue. Human, animals and environmental-related sources are indicated in dark blue, violet and light green circle, respectively.
