# Supplementary material for: Resistome Profiles, Plasmid Typing, and Whole-Genome Phylogenetic Tree Analyses of BlaNDM-9 and Mcr-1 Co-Harboring Escherichia coli ST617 from a Patient without a History of Farm Exposure in Korea
Source: Pathogens. 2019 Oct 31;8(4):212. doi: 10.3390/pathogens8040212 (PMC6963575; doi:10.3390/pathogens8040212)
Supplement: Supplementary file 1 [file pathogens-08-00212-s001.zip › Supplementary data Pathogen Figure S2.pdf]

Supplementary Data:

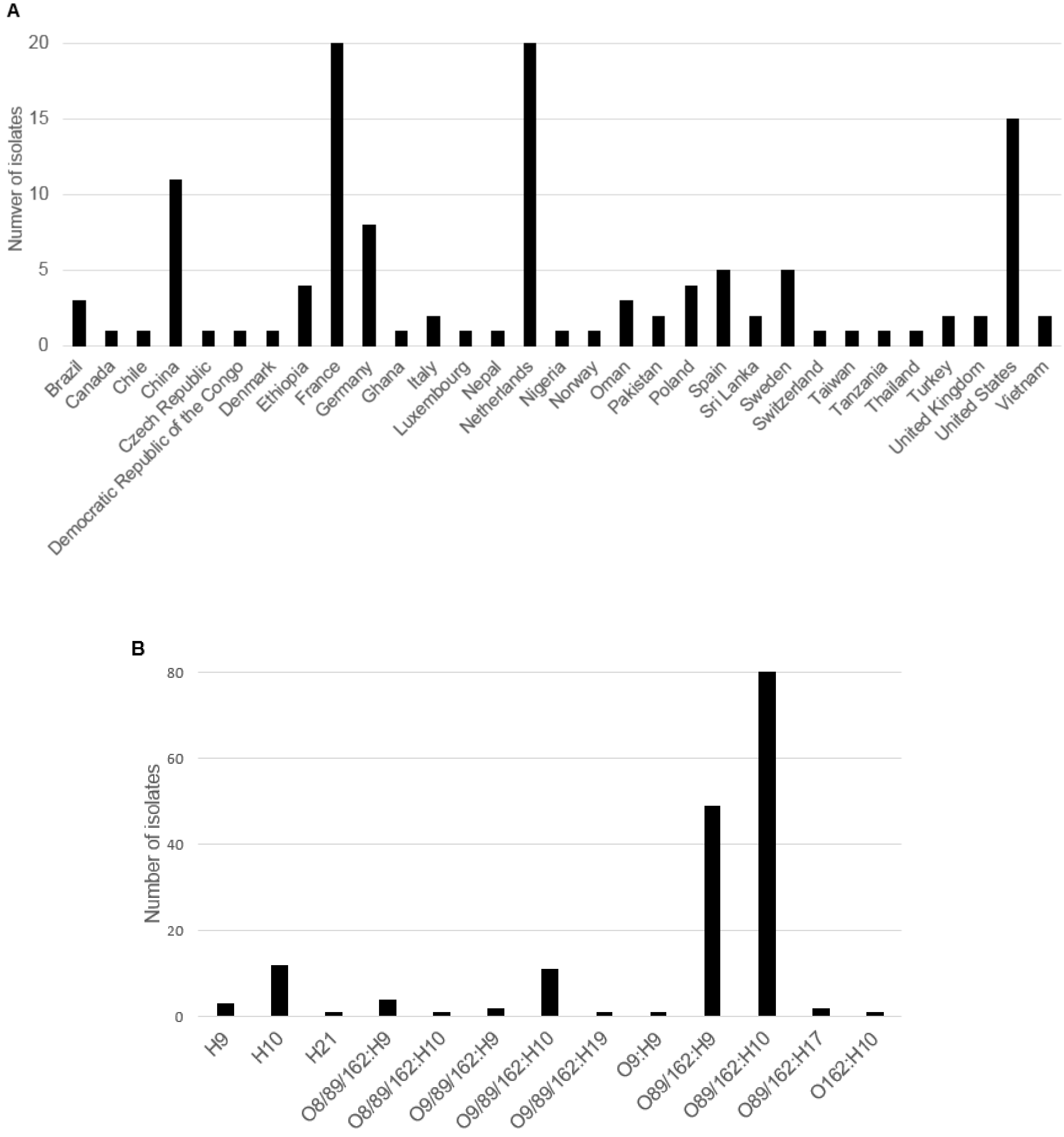

Figure S2. (A) Distribution of *E. coli* ST 617 among countries. (B) Distribution of serotypes among *E. coli* ST 617. All the data were collected and analyzed from EnteroBase (http://enterobase.warnick.ac.uk/)
