# Supplementary material for: Resistome Profiles, Plasmid Typing, and Whole-Genome Phylogenetic Tree Analyses of BlaNDM-9 and Mcr-1 Co-Harboring Escherichia coli ST617 from a Patient without a History of Farm Exposure in Korea
Source: Pathogens. 2019 Oct 31;8(4):212. doi: 10.3390/pathogens8040212 (PMC6963575; doi:10.3390/pathogens8040212)
Supplement: Supplementary file 1 [file pathogens-08-00212-s001.zip › Supplementary data Pathogen Table S1.pdf]

Supplementary Data:

Table S1. Primer sequences used for confirming the resistance genes and plasmid replicon types

Primer	Sequence (5’→3’)	Target length (bp)
bla$_{\text{NDM-9}}$_Fwd	AGGCGTTAGATTGGCTTAC	1207
bla$_{\text{NDM-9}}$_Rev	CATCGAGATCATCCAACCG	
bla$_{\text{CTX-M-55}}$_Fwd	CGGAAGGAGAACCAGGAAC	945
bla$_{\text{CTX-M-55}}$_Rev	CTGTTGTTAGGAAGTGTGCC	
bla$_{\text{TEM-1B}}$_Fwd	AGTGGGTTACATCGAACTGG	645
bla$_{\text{TEM-1B}}$ _Rev	ACGATACGGGAGGGCTTAC	
*mcr-1*_Fwd	GACGGCAAGATTCTTGAGGC	2165
*mcr-1*_Rev	CTCATCTCAGCAAGTAGGCG	
*aph(3')-Iia*_Fwd	GAACAAGATGGATTGCACGC	781
*aph(3')-Iia*_Rev	AGAACTCGTCAAGAAGGCG	
*aph(3’)Ib*_Fwd	ATCGCATTCTGACTGGTTGC	750
*aph(3’)Ib*_Rev	TCGCAGATAGAAGGCAAGGC	
*aph(6)-Id*_Fwd	AACCTGTTCTCATTGCGGAC	793
*aph(6)-Id*_Rev	TAGTATGACGTCTGTCGCAC	
*aadA2*_Fwd	CTTGATGATCTCGCCTTTCAC	763
*aadA2*_Rev	ACCATCGAAATTTCGAACCAAC	
*oqxA*_Fwd	ATCAGTTAAGGGTGGCGC	1177
*oqxA*_Rev	ATGAGCCTGCAAAAAACCTG	
*oqxB*_Fwd	CCAAACAGGCGATCGATCAG	1360
*oqxB*_Rev	GCTGGAGGAAGCGATCAAC	
*fosA3*_Fwd	GCTGAACTAACCCGTCAATC	379
*fosA3*_Rev	CCTGGCATTTTATCAGCAGT	
*mph(A)*_Fwd	ATGACCGTAGTCACGACCG	865
*mph(A)*_Rev	CTTCGTTACCCGAGTCGAG	
*mdf(A)*_Fwd	GGCCGTGGTGGAACAATATC	825
*mdf(A)*_Rev	CATCCATAAATACGCGTGCG	
*floR*_Fwd	TGATGGCTCCTTTCGACATC	1164
*floR*_Rev	GTTAGACGACTGGCGACTTC	
*sul2*_Fwd	ACCGCAAACAGGTTACTCGC	1168
*sul2*_Rev	GAGAAATATCCCGAATGTGCAG	
*tet(A)*_Fwd	TGTGCAACGGGAATTTGAAG	1259
*tet(A)*_Rev	AGAATCCGCGCGTTCAATC	
*dfrA12*_Fwd	GACGCGCATAAACGGAGTG	488
*dfrA12*_Rev	ATGAACTCGGAATCAGTACGC	
*rmtB*_Fwd	GCCATGGCACTTTTACAATC	730
*rmtB*_Rev	TATAAGTTCTGTTCCGATGGTC	
IncB_Fwd	TGAAAAACAGGGGGAGCAAG	1312
IncB_Rev	TGCAGTGGCTTTTGGGATC	
IncFII_Fwd	ATGTCGCAGACAGAAAATGC	1397
IncFII_Rev	CTGTAATTGCGGTTACGTGAC	
IncI2_Fwd	GTGTGAGTGGGTGTATGCAG	1467
IncI2_Rev	CTGTCAGGGCCAGTTCAAG	
IncN_Fwd	AGCAGGATCAAGGAAAGATCG	560
IncN_Rev	GCTGCTTACTACCTCTCGC	

IncY_Fwd	GTTTCCTTCCAACCGGCTTTG	1189
IncY_Rev	CCTCCAGCACACACTTATTCG	
IncR_Fwd	ACCGACTATTTGCAACAGTG	561
IncR_Rev	ATGGGAAAGAGGTCAAGTTC	
IncX1_Fwd	TCATTGCTGGCTTATCTCGC	477
IncX1_Rev	GTCCGTTTTGAGATCGAAATGAG	

3
